# The genome sequence of the Large Longhorn, *Nematopogon swammerdamella *(Linnaeus, 1758)

**DOI:** 10.12688/wellcomeopenres.20186.1

**Published:** 2023-11-17

**Authors:** William B.V. Langdon, Cass Baumberg, University of Oxford and Wytham Woods Genome Acquisition Lab, Darwin Tree of Life Barcoding collective, Wellcome Sanger Institute Tree of Life programme, Wellcome Sanger Institute Scientific Operations: DNA Pipelines collective, Tree of Life Core Informatics collective, Darwin Tree of Life Consortium

**Affiliations:** 1 University of Oxford, Oxford, England, UK

**Keywords:** Nematopogon swammerdamella, Large Longhorn, genome sequence, chromosomal, Lepidoptera

## Abstract

We present a genome assembly from an individual male *Nematopogon swammerdamella* (the Large Longhorn; Arthropoda; Insecta; Lepidoptera; Adelidae). The genome sequence is 699.5 megabases in span. Most of the assembly is scaffolded into 31 chromosomal pseudomolecules, including the Z sex chromosome. The mitochondrial genome has also been assembled and is 16.46 kilobases in length.

## Species taxonomy

Eukaryota; Metazoa; Eumetazoa; Bilateria; Protostomia; Ecdysozoa; Panarthropoda; Arthropoda; Mandibulata; Pancrustacea; Hexapoda; Insecta; Dicondylia; Pterygota; Neoptera; Endopterygota; Amphiesmenoptera; Lepidoptera; Glossata; Neolepidoptera; Heteroneura; Incurvarioidea; Adelidae; *Nematopogon*; *Nematopogon swammerdamella* (Linnaeus, 1758) (NCBI:txid753375).

## Background

The Longhorn moths (Adelidae family) are so named because their antennae are up to four times as long as the forewings. *Nematopogon swammerdamella*, the Large Longhorn, is the largest of the plain-coloured Longhorns in Britain, with a forewing length of 8 to 11 mm (wingspan 18–22 mm), and in this species the antennae are almost twice (females) and two-and-a-half times (males) the length of the forewings ( Sterling & Parsons, 2018).

*Nematopogon swammerdamella* is widespread in the western Palaearctic, extending into the eastern Palaearctic, from northern and eastern Ireland to southern Fenno-Scandinavia and the northern Mediterranean. There are relatively few records in eastern Europe and Italy. In the Atlantic Archipelago, it is common in England but less so in Scotland and Ireland ( GBIF Secretariat, 2023).

The habitat of the Large Longhorn is woodland and parks with deciduous trees. This moth is univoltine, and adults fly in May and June. Adult females oviposit eggs in plant stems. The larvae feed on dead leaves and decaying plant matter, and construct a portable case ( Sterling & Parsons, 2018). Older caterpillars live in a bivalved case on the ground; they hibernate twice and pupate inside the case ( Flemish Entomological Society, 2023).

The genome of the Large Longhorn, *Nematopogon swammerdamella*, was sequenced as part of the Darwin Tree of Life Project, a collaborative effort to sequence all named eukaryotic species in the Atlantic Archipelago of Britain and Ireland. Here we present a chromosomally complete genome sequence for *Nematopogon swammerdamella*, based on one male specimen from Wytham Woods, Oxfordshire, UK.

## Genome sequence report

**Figure 1.**
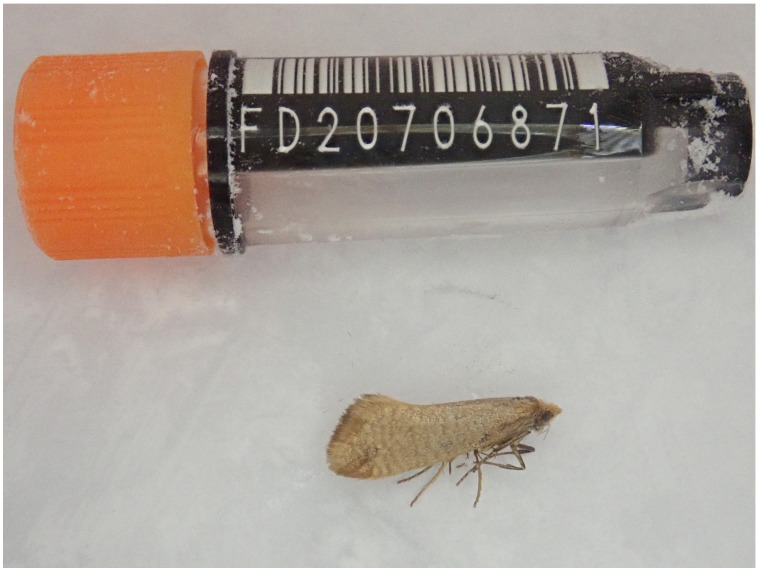
Photograph of the *Nematopogon swammerdamella*(ilNemSwae1) specimen used for genome sequencing.

The genome was sequenced from one male *Nematopogon swammerdamella* ( Figure 1) collected from Wytham Woods, Oxfordshire, UK (51.77, –1.34). A total of 37-fold coverage in Pacific Biosciences single-molecule HiFi long reads was generated. Primary assembly contigs were scaffolded with chromosome conformation Hi-C data. Manual assembly curation corrected 34 missing joins or mis-joins and removed 11 haplotypic duplications, reducing the assembly length by 0.81% and the scaffold number by 38.82%, and increasing the scaffold N50 by 1.54%.

**Figure 2.**
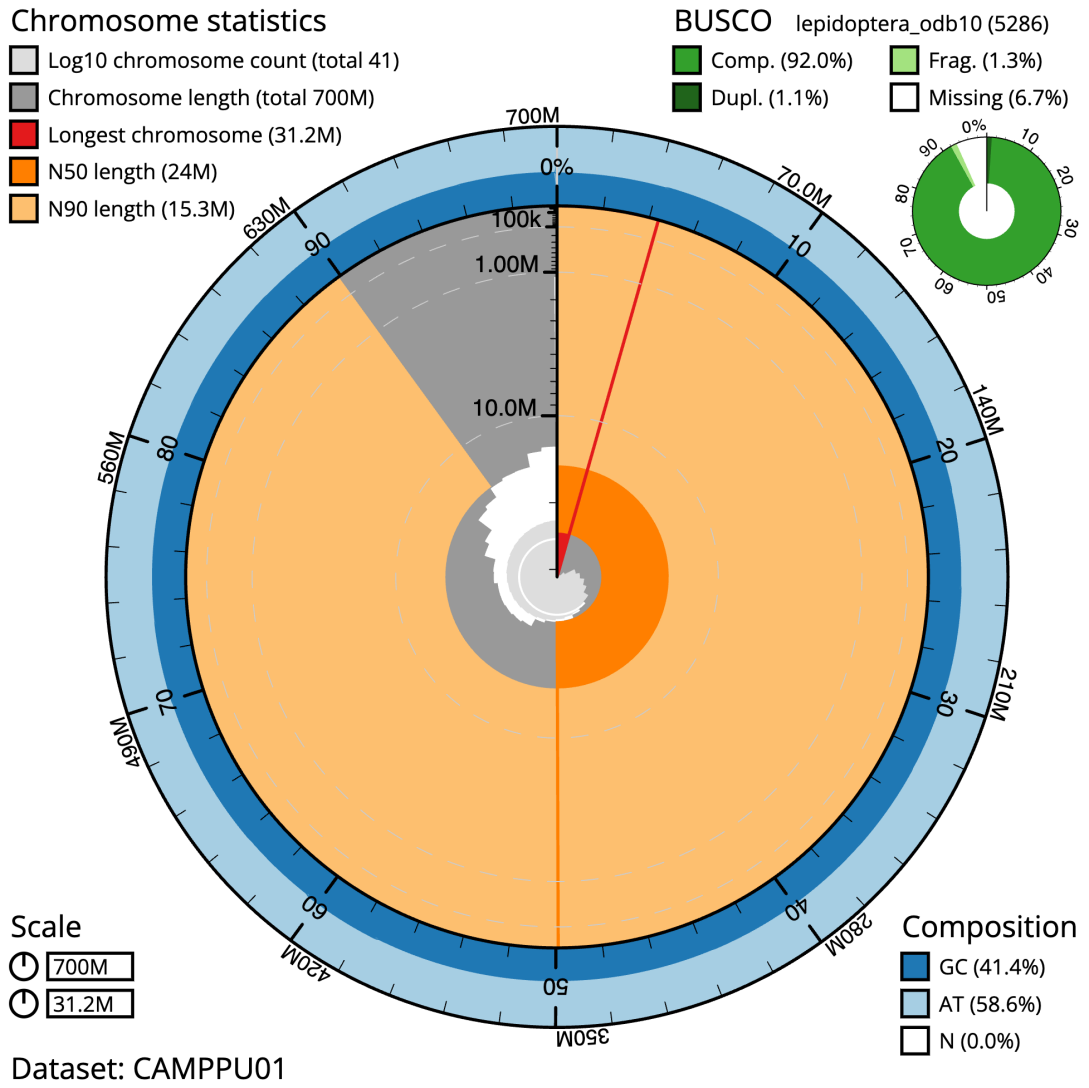
Genome assembly of *Nematopogon swammerdamella*, ilNemSwae1.1: metrics. The BlobToolKit Snailplot shows N50 metrics and BUSCO gene completeness. The main plot is divided into 1,000 size-ordered bins around the circumference with each bin representing 0.1% of the 699,520,461 bp assembly. The distribution of scaffold lengths is shown in dark grey with the plot radius scaled to the longest scaffold present in the assembly (31,243,614 bp, shown in red). Orange and pale-orange arcs show the N50 and N90 scaffold lengths (23,985,694 and 15,279,983 bp), respectively. The pale grey spiral shows the cumulative scaffold count on a log scale with white scale lines showing successive orders of magnitude. The blue and pale-blue area around the outside of the plot shows the distribution of GC, AT and N percentages in the same bins as the inner plot. A summary of complete, fragmented, duplicated and missing BUSCO genes in the lepidoptera_odb10 set is shown in the top right. An interactive version of this figure is available at https://blobtoolkit.genomehubs.org/view/ilNemSwae1.1/dataset/CAMPPU01/snail.

**Figure 3.**
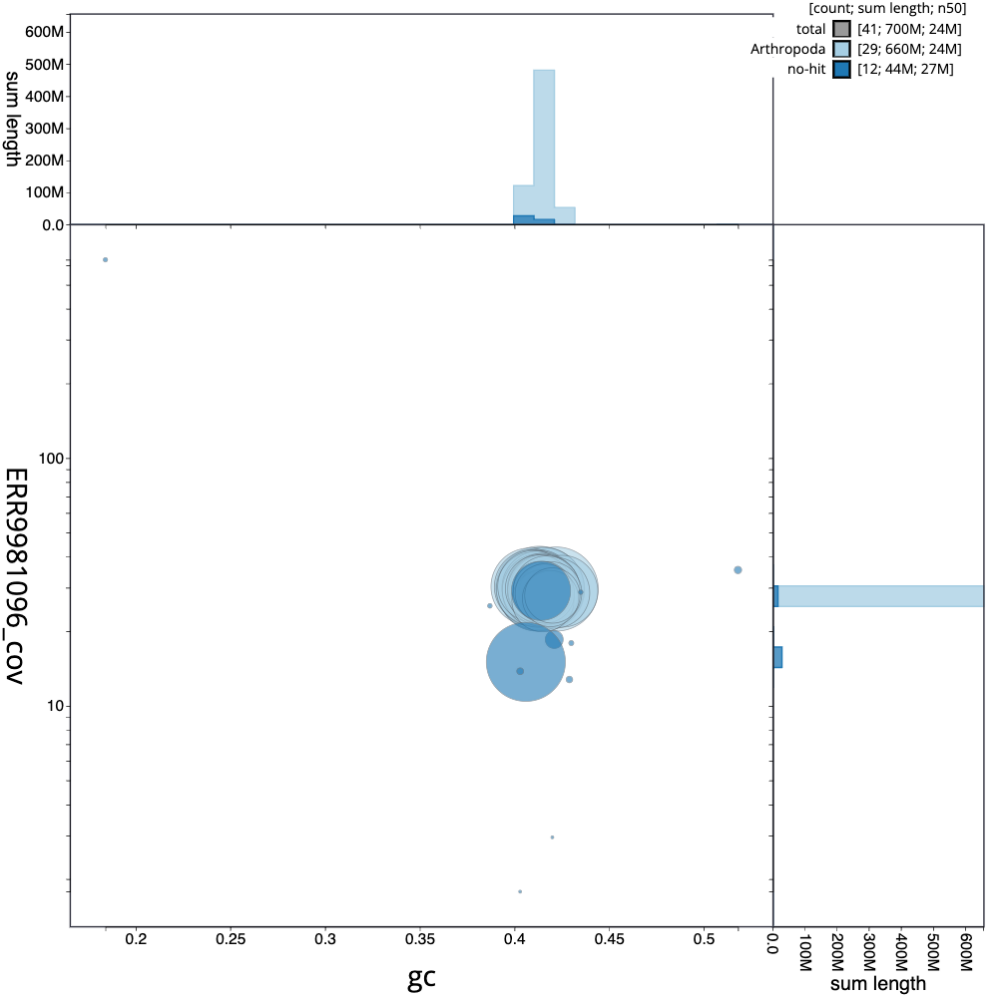
Genome assembly of *Nematopogon swammerdamella*, ilNemSwae1.1: BlobToolKit GC-coverage plot. Scaffolds are coloured by phylum. Circles are sized in proportion to scaffold length. Histograms show the distribution of scaffold length sum along each axis. An interactive version of this figure is available at https://blobtoolkit.genomehubs.org/view/ilNemSwae1.1/dataset/CAMPPU01/blob.

**Figure 4.**
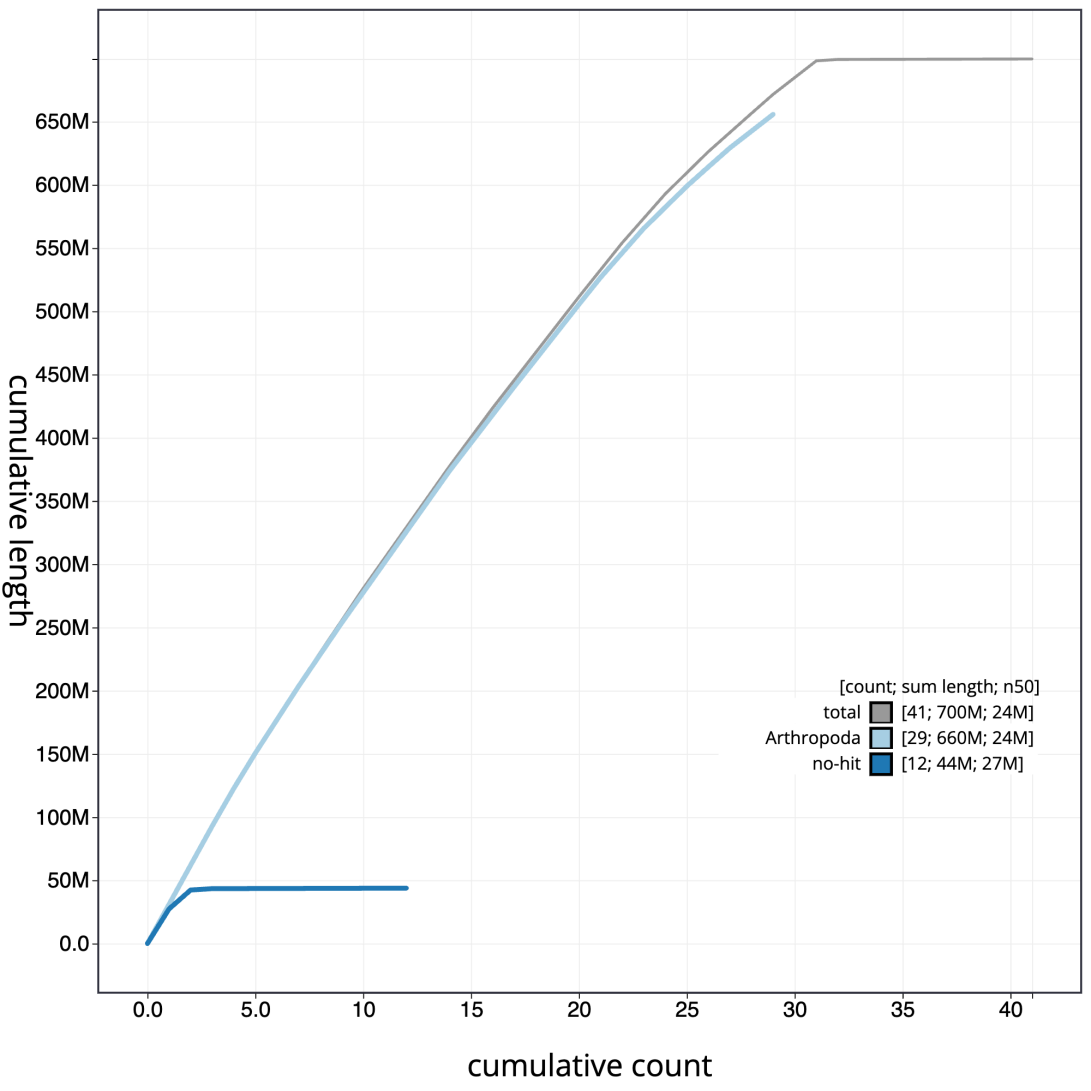
Genome assembly of *Nematopogon swammerdamella*, ilNemSwae1.1: BlobToolKit cumulative sequence plot. The grey line shows cumulative length for all scaffolds. Coloured lines show cumulative lengths of scaffolds assigned to each phylum using the buscogenes taxrule. An interactive version of this figure is available at https://blobtoolkit.genomehubs.org/view/ilNemSwae1.1/dataset/CAMPPU01/cumulative.

**Figure 5.**
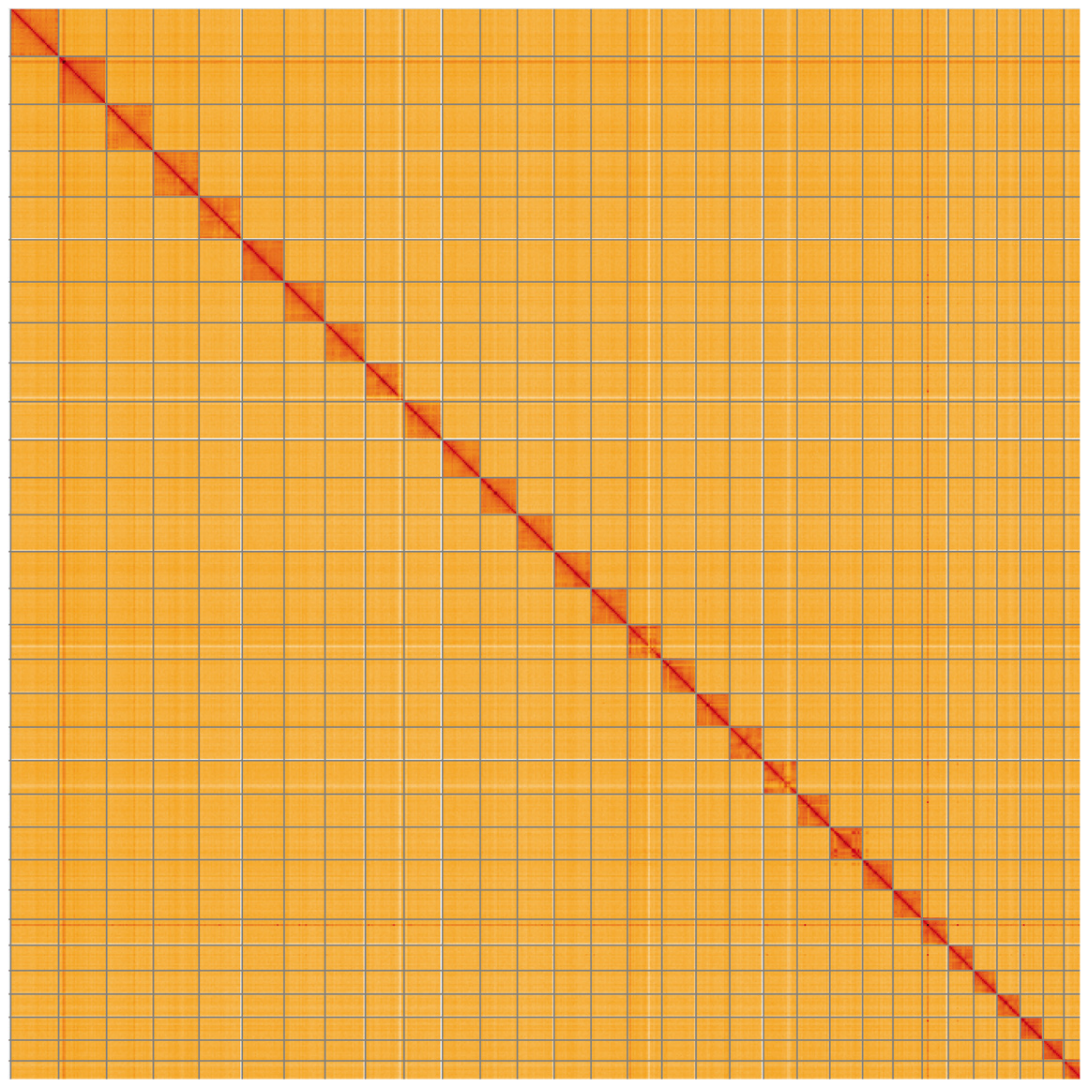
Genome assembly of *Nematopogon swammerdamella*, ilNemSwae1.1: Hi-C contact map of the ilNemSwae1.1 assembly, visualised using HiGlass. Chromosomes are shown in order of size from left to right and top to bottom. An interactive version of this figure may be viewed at https://genome-note-higlass.tol.sanger.ac.uk/l/?d=bZaoAHCdQ96v1XkBk4NOgg.

The final assembly has a total length of 699.5 Mb in 40 sequence scaffolds with a scaffold N50 of 24.0 Mb ( Table 1). A summary of the assembly statistics is shown in Figure 2, while the distribution of assembly scaffolds on GC proportion and coverage is shown in Figure 3. The cumulative assembly plot in Figure 4 shows curves for subsets of scaffolds assigned to different phyla. Most (99.97%) of the assembly sequence was assigned to 31 chromosomal-level scaffolds, representing 30 autosomes and the Z sex chromosome. Chromosome-scale scaffolds confirmed by the Hi-C data are named in order of size ( Figure 5; Table 2). While not fully phased, the assembly deposited is of one haplotype. Contigs corresponding to the second haplotype have also been deposited. The mitochondrial genome was also assembled and can be found as a contig within the multifasta file of the genome submission.

**Table 1.** Genome data for *Nematopogon swammerdamella*, ilNemSwae1.1.

Project accession data		
Assembly identifier	ilNemSwae1.1	
Species	*Nematopogon swammerdamella*	
Specimen	ilNemSwae1	
NCBI taxonomy ID	753375	
BioProject	PRJEB54807	
BioSample ID	SAMEA10166818	
Isolate information	ilNemSwae1, male: whole organism (DNA sequencing) ilNemSwae2, male: whole organism (Hi-C data)	
Assembly metrics *		*Benchmark*
Consensus quality (QV)	62.4	*≥ 50*
*k*-mer completeness	100%	*≥ 95%*
BUSCO **	C:92.0%[S:90.9%,D:1.1%], F:1.3%,M:6.7%,n:5,286	*C ≥ 95%*
Percentage of assembly mapped to chromosomes	99.97%	*≥ 95%*
Sex chromosomes	Z chromosome	*localised homologous pairs*
Organelles	Mitochondrial genome assembled	*complete single alleles*
Raw data accessions		
PacificBiosciences SEQUEL II	ERR9981096	
Hi-C Illumina	ERR9988138	
Genome assembly		
Assembly accession	GCA_946902875.1	
*Accession of alternate haplotype*	GCA_946902865.1	
Span (Mb)	699.5	
Number of contigs	258	
Contig N50 length (Mb)	5.5	
Number of scaffolds	40	
Scaffold N50 length (Mb)	24.0	
Longest scaffold (Mb)	27.4	

* Assembly metric benchmarks are adapted from column VGP-2020 of “Table 1: Proposed standards and metrics for defining genome assembly quality” from ( Rhie *et al.*, 2021). ** BUSCO scores based on the lepidoptera_odb10 BUSCO set using v5.3.2. C = complete [S = single copy, D = duplicated], F = fragmented, M = missing, n = number of orthologues in comparison. A full set of BUSCO scores is available at https://blobtoolkit.genomehubs.org/view/ilNemSwae1.1/dataset/CAMPPU01/busco.

**Table 2.** Chromosomal pseudomolecules in the genome assembly of *Nematopogon swammerdamella*, ilNemSwae1.

INSDC accession	Chromosome	Length (Mb)	GC%
OX336333.1	1	31.24	41.5
OX336334.1	2	31.11	42.0
OX336335.1	3	30.52	41.5
OX336336.1	4	29.72	41.5
OX336337.1	5	27.95	41.0
OX336339.1	6	26.66	41.0
OX336340.1	7	26.11	41.0
OX336341.1	8	25.29	41.0
OX336342.1	9	24.89	41.0
OX336343.1	10	24.57	41.5
OX336344.1	11	24.11	41.0
OX336345.1	12	23.99	41.0
OX336346.1	13	23.98	41.5
OX336347.1	14	23.62	41.5
OX336348.1	15	22.44	42.5
OX336349.1	16	22.17	41.0
OX336350.1	17	21.99	41.5
OX336352.1	19	21.87	42.0
OX336351.1	18	21.87	41.5
OX336353.1	20	21.36	41.5
OX336354.1	21	21.25	41.5
OX336355.1	22	19.7	41.5
OX336356.1	23	19.05	41.5
OX336357.1	24	16.92	42.0
OX336358.1	25	16.52	41.5
OX336359.1	26	15.28	41.5
OX336360.1	27	15.03	41.5
OX336361.1	28	14.98	41.5
OX336362.1	29	13.45	42.0
OX336363.1	30	13.04	42.0
OX336338.1	Z	27.35	40.5
OX336364.1	MT	0.02	19.0

The estimated Quality Value (QV) of the final assembly is 62.4 with *k*-mer completeness of 100%, and the assembly has a BUSCO v5.3.2 completeness of 92.0% (single = 90.9%, duplicated = 1.1%), using the lepidoptera_odb10 reference set ( *n* = 5,286).

Metadata for specimens, spectral estimates, sequencing runs, contaminants and pre-curation assembly statistics can be found at https://links.tol.sanger.ac.uk/species/753375.

## Methods

### Sample acquisition and nucleic acid extraction

Two male *Nematopogon swammerdamellai* were netted in Wytham Woods, Oxfordshire (biological vice-county Berkshire), UK (latitude 51.77, longitude –1.34) on 2021-05-11 by netting. The specimen was collected by Will Langdon (University of Oxford) and Cass Baumberg and identified by Will Langdon and preserved on dry ice. The specimen with ID Ox001344 (ToLID ilNemSwae1) was used for DNA sequencing, while the specimen with ID Ox001345 (ToLID ilNemSwae2) was used for Hi-C scaffolding. The species identification was confirmed by DNA barcoding.

The ilNemSwae1 sample was prepared for DNA extraction at the Tree of Life laboratory, Wellcome Sanger Institute (WSI). The sample was weighed and dissected on dry ice with tissue set aside for Hi-C sequencing. Tissue from the whole organism was disrupted using a Nippi Powermasher fitted with a BioMasher pestle. DNA was extracted at the Wellcome Sanger Institute (WSI) Scientific Operations core using the Qiagen MagAttract HMW DNA kit, according to the manufacturer’s instructions.

### Sequencing

Pacific Biosciences HiFi circular consensus DNA sequencing libraries were constructed according to the manufacturers’ instructions. DNA sequencing was performed by the Scientific Operations core at the WSI on a Pacific Biosciences SEQUEL IIe (HiFi) instrument. Hi-C data were also generated from whole organism tissue of ilNemSwae2 using the Arima2 kit and sequenced on the Illumina NovaSeq 6000 instrument.

### Genome assembly, curation and evaluation

Assembly was carried out with Hifiasm ( Cheng *et al.*, 2021) and haplotypic duplication was identified and removed with purge_dups ( Guan *et al.*, 2020). The assembly was then scaffolded with Hi-C data ( Rao *et al.*, 2014) using YaHS ( Zhou *et al.*, 2023). The assembly was checked for contamination and corrected as described previously ( Howe *et al.*, 2021). Manual curation was performed using HiGlass ( Kerpedjiev *et al.*, 2018) and Pretext ( Harry, 2022). The mitochondrial genome was assembled using MitoHiFi ( Uliano-Silva *et al.*, 2023), which runs MitoFinder ( Allio *et al.*, 2020) or MITOS ( Bernt *et al.*, 2013) and uses these annotations to select the final mitochondrial contig and to ensure the general quality of the sequence.

A Hi-C map for the final assembly was produced using bwa-mem2 ( Vasimuddin *et al.*, 2019) in the Cooler file format ( Abdennur & Mirny, 2020). To assess the assembly metrics, the *k*-mer completeness and QV consensus quality values were calculated in Merqury ( Rhie *et al.*, 2020). This work was done using Nextflow ( Di Tommaso *et al.*, 2017) DSL2 pipelines “sanger-tol/readmapping” ( Surana *et al.*, 2023a) and “sanger-tol/genomenote” ( Surana *et al.*, 2023b). The genome was analysed within the BlobToolKit environment ( Challis *et al.*, 2020) and BUSCO scores ( Manni *et al.*, 2021; Simão *et al.*, 2015) were calculated.

Table 3 contains a list of relevant software tool versions and sources.

**Table 3.** Software tools: versions and sources.

Software tool	Version	Source
BlobToolKit	4.0.7	https://github.com/blobtoolkit/blobtoolkit
BUSCO	5.3.2	https://gitlab.com/ezlab/busco
Hifiasm	0.16.1-r375	https://github.com/chhylp123/hifiasm
HiGlass	1.11.6	https://github.com/higlass/higlass
Merqury	MerquryFK	https://github.com/thegenemyers/MERQURY.FK
MitoHiFi	2	https://github.com/marcelauliano/MitoHiFi
PretextView	0.2	https://github.com/wtsi-hpag/PretextView
purge_dups	1.2.3	https://github.com/dfguan/purge_dups
sanger-tol/genomenote	v1.0	https://github.com/sanger-tol/genomenote
sanger-tol/readmapping	1.1.0	https://github.com/sanger-tol/readmapping/tree/1.1.0
YaHS	yahs-1.1.91eebc2	https://github.com/c-zhou/yahs

### Wellcome Sanger Institute – Legal and Governance

The materials that have contributed to this genome note have been supplied by a Darwin Tree of Life Partner. The submission of materials by a Darwin Tree of Life Partner is subject to the **‘Darwin Tree of Life Project Sampling Code of Practice’**, which can be found in full on the Darwin Tree of Life website here. By agreeing with and signing up to the Sampling Code of Practice, the Darwin Tree of Life Partner agrees they will meet the legal and ethical requirements and standards set out within this document in respect of all samples acquired for, and supplied to, the Darwin Tree of Life Project. 

Further, the Wellcome Sanger Institute employs a process whereby due diligence is carried out proportionate to the nature of the materials themselves, and the circumstances under which they have been/are to be collected and provided for use. The purpose of this is to address and mitigate any potential legal and/or ethical implications of receipt and use of the materials as part of the research project, and to ensure that in doing so we align with best practice wherever possible. The overarching areas of consideration are:

• Ethical review of provenance and sourcing of the material

• Legality of collection, transfer and use (national and international) 

Each transfer of samples is further undertaken according to a Research Collaboration Agreement or Material Transfer Agreement entered into by the Darwin Tree of Life Partner, Genome Research Limited (operating as the Wellcome Sanger Institute), and in some circumstances other Darwin Tree of Life collaborators.

## Data Availability

European Nucleotide Archive: *Nematopogon swammerdamella* (large long-horn). Accession number PRJEB54807; https://identifiers.org/ena.embl/PRJEB54807. ( Wellcome Sanger Institute, 2022) The genome sequence is released openly for reuse. The *Nematopogon swammerdamella* genome sequencing initiative is part of the Darwin Tree of Life (DToL) project. All raw sequence data and the assembly have been deposited in INSDC databases. The genome will be annotated using available RNA-Seq data and presented through the Ensembl pipeline at the European Bioinformatics Institute. Raw data and assembly accession identifiers are reported in Table 1.

## Author information

Members of the University of Oxford and Wytham Woods Genome Acquisition Lab are listed here: https://doi.org/10.5281/zenodo.4789928.

Members of the Darwin Tree of Life Barcoding collective are listed here: https://doi.org/10.5281/zenodo.4893703.

Members of the Wellcome Sanger Institute Tree of Life programme are listed here: https://doi.org/10.5281/zenodo.4783585.

Members of Wellcome Sanger Institute Scientific Operations: DNA Pipelines collective are listed here: https://doi.org/10.5281/zenodo.4790455.

Members of the Tree of Life Core Informatics collective are listed here: https://doi.org/10.5281/zenodo.5013541.

Members of the Darwin Tree of Life Consortium are listed here: https://doi.org/10.5281/zenodo.4783558.
