# Supplementary material for: The genome sequence of the Large Longhorn, Nematopogon swammerdamella (Linnaeus, 1758)
Source: Wellcome Open Res. 2023 Nov 17;8:531. [Version 1] doi: 10.12688/wellcomeopenres.20186.1 (PMC11372353; doi:10.12688/wellcomeopenres.20186.1)
Supplement: Peer review history

# Reviewer report: Reviewer response for version 1

**DOI:** 10.21956/wellcomeopenres.22349.r78494

**Published:** 2024-09-03

**Authors:** Beatriz Willink

**Affiliations:** 1 Department of Biological Sciences, National University of Singapore (Ringgold ID: 37580), Singapore, Singapore; 2 Department of Zoology, Stockholm University, Stockholm, Sweden

This data note describes an assembly for the Large Longhorn moth *Nematopogon swammerdamella.* At the time of this review, there are only two genome assemblies for the Adelidae moth family, and this is the only one with chromosome level contiguity. I believe this new assembly could be of great importance for future comparative, evolutionary and phylogenomic studies in Lepidoptera, given the early divergence of Adelidae (incl. Nematopogon) from Ditrysia, the clade currently comprising the vast majority (>95%) of Lepidoptera species.

 The authors used high quality data and clearly written methods to generate this assembly. Two notable strengths are the abundant coverage of long-read sequence data (37X) and the use of additional chromatin interaction data, which together allow for near chromosome-level resolution. The raw data, main assembly, and haplotigs are available online as stated. I found no major weaknesses in the paper, but highlight here a few of minor issues to clarify or expand:

 1. For the reasons mentioned above, I think that the Introduction would benefit from a short overview of the phylogenetic context of the taxon. The references below are two recent phylogenetic studies looking at the early evolution of Lepidoptera, and thus include information about the relationships between Adelidae and other early-diverging Lepidoptera lineages.

 2. I was surprised to see the relatively high number of missing BUSCOs (6.7%), compared to other assemblies with similar sequencing coverage. Without being an expert in this clade, I presume the the set of conserved Lepidoptera genes in lepidoptera_odb10 come primarily for studies in Dytrisia, which encompasses the vast majority of species (and research) in Lepidoptera. I thus wonder if some of these “missing” genes, partly indicate a assembly completeness, but also could tell us about genes that evolved in Dytrisia, and are therefore absent in *Nematopogon. *I’m not suggesting to include a comparative analysis here, but I think, once again given the ancestral origin of this clade, that the relative large fraction of “missing” genes is noteworthy.

 3. Was MitoHiFi run with MitoFinder, MITOS, or both? This is not entirely clear in the methods?

 4. The accessions for the individual chromosomes are provided in Table 2, but I’d suggest adding the accession links for the main assembly and haplotigs.

 5. Pleas clarify how the Z chromosome was identified.

Are sufficient details of methods and materials provided to allow replication by others?

Partly

Is the rationale for creating the dataset(s) clearly described?

Yes

Are the datasets clearly presented in a useable and accessible format?

Yes

Are the protocols appropriate and is the work technically sound?

Yes

Reviewer Expertise:

evolution of sex-limited polymorphisms in insects

I confirm that I have read this submission and believe that I have an appropriate level of expertise to confirm that it is of an acceptable scientific standard.

## References

[1] : The unresolved phylogenomic tree of butterflies and moths (Lepidoptera): Assessing the potential causes and consequences. *Systematic Entomology* .2022;47(4) : 10.1111/syen.12545 531-550 10.1111/syen.12545

[2] : Higher-level phylogeny and evolutionary history of nonditrysians (Lepidoptera) inferred from mitochondrial genome sequences. *Zoological Journal of the Linnean Society* .2023;198(2) : 10.1093/zoolinnean/zlac096 476-493 10.1093/zoolinnean/zlac096

# Reviewer report: Reviewer response for version 1

**DOI:** 10.21956/wellcomeopenres.22349.r70476

**Published:** 2024-05-11

**Authors:** Jurate De Prins

**Affiliations:** 1 Royal Belgian Institute of Natural Sciences, Brussels, Belgium

It is a great pleasure to see one more successful trial to study the full genomes of European Microlepidoptera. I am very pleased that this approach gets the needed attention and support. Microlepidoptera have a very long and complex evolutionary history, many of them are narrow host-specific and are external or internal feeders of different parts of plants. Therefore, they can be dispersed very easily to all parts of the world by human activities. The long-sequence genetic information, obtained from the reliably identified Microlepidoptera species is a needed step forward and I congratulate the authors for doing so. This is the major positive point of the article, and that's why this manuscript is approved by me .

 I am not a molecular specialist, my background is in karyology and taxonomy, therefore, I will touch on those aspects of the article with deeper insight. 

 No doubt that studying the full genomes of European microlepidoptera species is a long time-consuming and financial commitment. There is one very strong demand from the community of researchers and from future generations of entomologists that the identification of species should be correct. In this particular case, *Nematopogon swammerdamella* (Linnaeus, 1758) is identified correctly, however, in the majority of cases, Microlepidoptera specimens are identified not only from the external characters but from internal characteristics of male and female genitalia. Therefore, the management of the project should ensure the community that the identification of samples is correct.

 Very happy that the Tree of Life Consortium started to pay attention to the chromosomes. I worked on Lepidoptera chromosomes many years ago and learned what a complex evolutionary history they passed until we see the present pattern. 

 Very happy that my article published in 2001 was recently cited by the team of the Tree of Life in top quality journal Nature Ecology and Evolution see https://www.nature.com/articles/s41559-024-02329-4

 The sex mechanism ZZ or Z0 is important for interspecific possible hybridization, and I hope that now when full genomes are available the authors will clarify it in the future.

Are sufficient details of methods and materials provided to allow replication by others?

Yes

Is the rationale for creating the dataset(s) clearly described?

Yes

Are the datasets clearly presented in a useable and accessible format?

Yes

Are the protocols appropriate and is the work technically sound?

Yes

Reviewer Expertise:

Taxonomy, systematics, biodiversity data management

I confirm that I have read this submission and believe that I have an appropriate level of expertise to confirm that it is of an acceptable scientific standard.

## References

[1] Reference source

# Reviewer report: Reviewer response for version 1

**DOI:** 10.21956/wellcomeopenres.22349.r78500

**Published:** 2024-04-26

**Authors:** Chen Wu

**Affiliations:** 1 The New Zealand Institute of Plant and Food Research Limited, Auckland, New Zealand

Summary: paper described a male *Nematopogon swammerdamellari* genome assembly sequenced and assembled from HiFi data. The assembly was built from typical HiFi assembler and assessed quality using typical quality qc tools. However, there are a few things required to be put in detail.

 1. paper described this is a male genome assembly including the Z chromosome assembled in the abstract, but no detail regarding to how they identified Z chromosome from the scaffold pool and what it looks like. I feel some sex chromosome descriptions are also required in the background session.

 2. I found it is hard to reproduce with method description, such as parameters (whether default settings or with additional option alterations).

 3. Some raw data information are missing, such as Kmer spectrum and long-read length distribution. what does '37-fold coverage' come from? do you have genome size estimated using some methods?

 4. paper only reports primary assembly, but Hifiasm is able to produce two haplotype assemblies as well. It would be better to include some descriptions and release of those, which might be useful for other researchers in future.

 5. Would be good to have a synteny analysis with a closely related genome

Are sufficient details of methods and materials provided to allow replication by others?

No

Is the rationale for creating the dataset(s) clearly described?

Partly

Are the datasets clearly presented in a useable and accessible format?

Yes

Are the protocols appropriate and is the work technically sound?

Yes

Reviewer Expertise:

bioinformatics, genomics, transcriptomics

I confirm that I have read this submission and believe that I have an appropriate level of expertise to confirm that it is of an acceptable scientific standard, however I have significant reservations, as outlined above.
